# Epigenome: A Biomarker or Screening Tool to Evaluate Health Impact of Cumulative Exposure to Chemical and Non-Chemical Stressors

**DOI:** 10.3390/bios6020012

**Published:** 2016-04-01

**Authors:** Kenneth Olden, Yu-Sheng Lin, David Bussard

**Affiliations:** National Center for Environmental Assessment, Office of Research and Development, U.S. Environmental Protection Agency, Washington, DC 20460, USA; Lin.Yu-Sheng@epa.gov (Y.-S.L.); bussard.david@epa.gov (D.B.)

**Keywords:** chemical, cumulative, DNA methylation, environmental, epigenetics, epigenome, non-chemical, histone modification, stressors

## Abstract

Current risk assessment practices and toxicity information are hard to utilize for assessing the health impact of combined or cumulative exposure to multiple chemical and non-chemical stressors encountered in the “real world” environment. Non-chemical stressors such as heat, radiation, noise, humidity, bacterial and viral agents, and social factors, like stress related to violence and socioeconomic position generally cannot be currently incorporated into the risk assessment paradigm. The Science and Decisions report released by the National Research Council (NRC) in 2009 emphasized the need to characterize the effects of multiple stressors, both chemical and non-chemical exposures. One impediment to developing information relating such non-chemical stressors to health effects and incorporating them into cumulative assessment has been the lack of analytical tools to easily and quantitatively monitor the cumulative exposure to combined effects of stressors over the life course.

## 1. Background

Progress in environmental health research has greatly expanded our understanding of the role of the environment in human health and disease. There is now broad consensus that where people live and work makes a difference in their health; that is, neighborhood characteristics or place matters for human health and disease (just as in early days of epidemiology there were striking examples of how one’s occupation mattered for life expectancy). For example, the variation in life expectancy (a common measure of population health) between populations living in different neighborhoods (advantaged *versus* disadvantaged) in New Orleans, LA varies by as much as 25 years. Individuals living in the zip code 70124 have a life expectancy of 80 years compared to that of 55 years for individuals who live in zip code 70112. These two neighborhoods are separated by less than three miles [1]. Similar variation in life expectancy has been reported for other cities (e.g., New York City), and for the U.S. at large [2]. The latter landmark study concluded that the most significant risk factors accounting for the regional differential in health outcomes are related to the environment [3].

Nevertheless, the complex relationship between environmental exposure and disease remains poorly understood. Exactly why neighborhood matters for human health and disease is largely unknown. However, it is widely accepted that neighborhood differences in life expectancy are related to poverty, lifestyle, and regional variation in exposure to toxic chemical and other non-chemical stressors [2,3]. The mechanisms by which these factors operate are less clear. And, for any particular community, identification of the driving factors in that community can be difficult to identify. To a large extent, contributing factors are associated with choices in our public policies (e.g., housing, education, enforcement of environmental regulations, and access to healthcare); therefore, increase in life expectancy can be achieved, reminiscent of our success in controlling infectious diseases, by the adoption of public health measures.

It is unlikely that differences in genetic susceptibility are playing a significant role in health disparities between neighborhoods, even if playing a role in exactly which individuals develop a given disease. While candidate gene and genome wide association studies (GWAS) have identified multiple loci involved in the development of non-communicable diseases, genetic variation accounts for only a small fraction of the common diseases (e.g., coronary heart disease and diabetes) responsible for neighborhood differences in health outcomes [4,5]. However, advances in understanding the genetic contributions to common diseases will help advance the understanding of the neighborhood differences contributing to disease because studies of the roles of specific genes will also inform the linking of specific epigenetic changes to particular diseases. Epigenetics and gene expression studies coupled with exposure and location information then can relate specific epigenetic changes to particular environmental exposures.

The accurate measurement of environmental exposure remains an un-met need and existing methodologies do not match gene discovery with respect to rigor and precision. Therefore, in 2012, the National Institute of Health announced an initiative—the Exposome Project—to develop new tools to measure environmental exposure (http://www.niehs.nih.gov/about/strategicplan/index.cfm). The goal was to measure all exposures (both chemical and non-chemical) of an individual over the life course and relate the exposure to health. The success of this project is critically dependent on the development of analytic tools to accurately measure exposure. To date, the technology of choice to measure exposure has been metabolomics [6,7]. DNA and protein microarray have also shown considerable promise as screening tools for discovery of biomarkers of exposure [8,9]. The recent explosion of research in epigenetics has shown that environmental exposure to both chemical and non-chemical stressors leave durable, environment-specific, “imprints” on the epigenome [10,11,12]. Some of these persist long after the stressor is gone; some of these are more transient. The “imprints” are expressed in the form of changes such as DNA methylation, chemical modification of histones, and the expression of non-coding RNA [13,14,15]. We hypothesized that the imprinted epigenome can serve as a screening tool or biomarker of exposure to establish the plausibility of exposure-disease associations, and/or to serve as a biomarker of effect when coupled with transcriptomics, metabolomics, and metabolic pathway analyses [16,17,18]. However, the ability to link response to specific exposures may require specific exposure data and appropriate statistical methods to rigorously characterize neighborhoods with respect to environmental exposures.

Olden *et al.* (2011, 2014) [17,19] earlier suggested that advantaged and disadvantaged neighborhoods differentially “imprint” the epigenome, and these durable modifications can be detected as an exposure signature in population studies to assess cumulative impact of lifetime exposures.

The objective of this commentary is to explore the plausibility that environment-induced epigenetic signatures can be used as a biomarker to assess the combined or cumulative exposure to multiple chemical and non-chemical stressors over the life course. We surveyed the literature to determine whether studies exist to support the conclusion that epigenetic signatures satisfy the requirements for such a biomarker.

The plausibility of employing the epigenome as a biomarker of cumulative exposure is supported by the following experimental observations: First, a genome-wide examination of epigenetic modifications can detect changes within the same individual following exposure to a variety of environmental agents (e.g., chemicals, psychological stress, behavior, and socioeconomic position) [12,20,21,22]. Because different markers have different patterns of persistence, a genome-wide examination from an individual should be able to identify all epigenetic changes if there were exposures early in life or whether there were significant exposures recently. Also, some epigenetic markers may change progressively in degree and yield information about the cumulative exposure over time to particular stressors (e.g., prenatal exposure to maternal second-hand smoking) [23,24]. Second, most, if not all, diseases are associated with epigenetic changes consistent with their involvement in both their initiation and progression [25,26,27]. Therefore, some changes will be early markers of potential future disease and increased current sensitivity while others will be markers of a more advanced disease state with implications for appropriate intervention as well as further information on the likely time of prior stresses. Finally, both differential DNA methylation and increase in rate of chronic diseases are associated with aging [28].

Furthermore, studies show that epigenetic modifications are durable [29,30], exposure specific (e.g., acetaldehyde and cigarette smoking) [23,31,32], correlates with intensity of exposure [33,34], precedes histological/pathological evidence of disease [19,35,36], and alters metabolic pathways involved in the etiology of specific disease (e.g., Phthalate-induced glucometabolic dysfunction) [37,38]. For example, Joubert *et al.* (2012) reported that maternal smoking during pregnancy was associated with methylation of genes (CYP1A1 and AHRR) involved in the metabolism of carcinogens (e.g., polycyclic aromatic hydrocarbons) that play a role in the development of lung cancer [23].

Also, many epigenetic modifications can be detected in human tissues using minimally-invasive, low cost, and potentially high throughput technology. Markers are likely to differ in terms of their persistence and whether they provide information on long-term exposure or only recent exposures. The question of whether methylation measured in the blood is informative for organ- or tissue-specific diseases is still unsettled. Nevertheless, several studies suggest that blood is a useful surrogate tissue [39,40,41,42,43]. For example, Talens *et al.* (2010) compared epigenetic profiles of candidate loci in blood and buccal cells and found that DNA methylation was the same for more than half of the sites in both tissues. Likewise, the question of whether epigenetic markers are stable enough to be used in prospective cohort studies has been examined. It was found that several loci in both blood and buccal cell samples, repeatedly collected from the same individuals (age 14–62 years old) over a period of up to 20 years, generally remain stable [39].

Some markers, such as changes in the methylation of certain genes (e.g., AHRR) reflect exposures early in life and persist long into later life (e.g., infants exposed to second-hand smoking) [23,44]. Other markers, such as histone modifications or the expressions of noncoding RNA, seem to be more transient. While this complicates the formation of broad generalizations about epigenetic changes, it also creates opportunities for investigators to study the effects of all different types of epigenetic modifications, and by doing so, obtain information about likely exposures at different times and life stages—some indicating whether exposures occurred or not in early life exposures, some indicating there have been recent exposures, and some perhaps increasing in their degree of change as concentration and duration increases (as epidemiologists often phrase “cumulative exposure” discussing a single factor over time) [45].

## 2. Public Health Significance

Many of the differences in disease patterns between human populations might be mediated through, and detectable in, environment-induced epigenetic programming of gene expression. If this is the case, exciting opportunities exist to both prevent and treat common diseases. Identification of communities with elevated incidence of epigenetic changes related to important diseases can help target health prevention efforts. If epigenetic markers can be developed that help identify the key contributors to high disease risks in a community, these could allow even more focused intervention on the most important risk factors. Where factors can be reduced, such as if there is controllable exposure to harmful chemicals, the risk of adverse health outcomes could be reduced. Information that specific environmental exposures make a community particularly susceptible might be useful when setting regulatory standards or taking non-regulatory actions. Where specific risk factors cannot easily be reduced, strategies to limit the health impact or reduce other factors that contribute to the same disease but can be more easily modified, such as improved nutrition or exercise, could be motivated by specific information on the risks in the community. Potentially, perhaps many years out, one can use gene editing technology to restore normal gene function by reprogramming epigenetic mechanisms. Gene reprogramming can also be achieved using environmental or pharmaceutical intervention, since genes inactivated by epigenetic mechanisms are still intact—unlike those inactivated by mutations [46].

## 3. Conclusions

The findings discussed above are exciting in that they suggest that neighborhood-specific epigenome analysis may provide an excellent experimental model to examine the potential utility of epigenetic signatures as biomarker of cumulative exposure to chemical and non-chemical stressors. Given the nearly infinite number of combinations of chemical and non-chemical stressors that humans encounter in the environment over the life course, risk cannot be evaluated using traditional approaches. Furthermore, the effect of epigenetic modifications on gene expression and metabolic pathways might provide mechanistic insight to explain the strong linkage between neighborhood and life expectancy, and if our hypothesis is correct that differences in disease patterns between human populations are mediated by neighborhood- induced epigenetic programming of gene expression, this points to promising avenues for using epigenetic markers to better understand the health status of a community, the risk factors most contributing to that health status, and ways to target prevention and treatment of the common diseases elevated within a community as discussed above.

Some epigenetic changes are associated with both a stressor and a disease, perhaps serving as both markers of exposure and of effect. Over time, markers that are more “specific”, *i.e.*, only arise in response to certain exposures, will prove increasingly useful to identify a specific exposure that contributed to disease in a community. Markers that relate to a disease but are less specific to a particular exposure or stress, will prove to be integrating tools to assess the current health status and expected future susceptibility or risk of individuals in a community.

Even though the concept of the environment was expanded to include non-chemical stressors by the National Institute of Environmental Health Sciences (NIEHS) in the early 1990’s, they have not been integrated into the risk assessment-risk management paradigm, as suggested in the 2009 National Research Council (NRC) Report [47]. This has not occurred because the analysis of the relative quantitative contribution of different factors has often not been done and the tools for studying such interactions have been limited. However, with the emergence of epigenetics, we may now have the capacity to develop tools both to speed the epidemiological studies of how different stressors contribute to the magnitude of response to controllable stressors, and then to integrate community characteristics into the risk assessment-risk management paradigm and into community public health intervention and treatment programs.

Epigenome analysis may provide the best experimental tool for examining the potential sources of striking patterns in public health outcomes and a setting in which to test the utility of epigenetic signatures as biomarkers of exposure and effects [19]. However, prior to the full incorporation of epigenome analysis into the context of cumulative risk assessment, it may require a more fundamental understanding of the complex interactions between specific epigenetic changes, the environment and chronic disease as a roadmap for the future direction of efforts.

Our optimism about the potential of epigenetics is not to minimize the scientific work required to bring the ideas to practical scientific application. There are complexities to be investigated and challenges to overcome. For example, some epigenetic changes (e.g., the growth factor gene like insulin-like growth factor 2, IGF2) occur primarily during specific stages of development [48,49] whereas others (e.g., arsenic-related promoter hypermethylation of tumor suppressor genes such as p53) may occur in response to conditions throughout life [12,50]. Some changes of biological importance may only occur in tissues that are difficult to sample in humans [11,51]. Field studies in humans will face the kinds of complexities always faced in epidemiology of obtaining cooperation and records for a sizable cohort, and collecting and analyzing data on potential confounders [52]. Unravelling this will take time and creativity.

The payoff of such scientific investigation over time, however, could be significant in terms of scientific understanding and in terms of eventually providing tools for the efficient investigation of communities at risk to exposures of concern, and changes in patterns of disease.

If we can better understand the conditions under which epigenetic changes occur and either revert or persist, we will have a valuable tool to evaluate the interaction of environmental exposures and stresses with inherited genetics (and inherited epigenetics) and to identify adverse outcome pathways. This will uniquely provide a mechanistic tool to relate the contribution of environmental factors to human health and chronic disease.

## Abbreviations

AHRR	AhR repressor
CYP1A1	cytochrome P450 1A1
DNA	deoxyribonucleic acid
GWAS	genome wide association studies
IGF2	insulin-like growth factor 2
NIEHS	National Institute of Environmental Health Sciences
NRC	National Research Council
RNA	ribonucleic acid
